# Potential of cereal-based agricultural residues available for bioenergy production

**DOI:** 10.1016/j.dib.2019.103829

**Published:** 2019-03-16

**Authors:** Lisandra Rocha-Meneses, Thaísa Fernandes Bergamo, Timo Kikas

**Affiliations:** a Institute of Technology, Chair of Biosystems Engineering, Estonian University of Life Sciences, Kreutzwaldi 56, 51006, Tartu, Estonia; b Institute of Agricultural and Environmental Sciences, Chair of Environmental Protection and Landscape Management, Estonian University of Life Sciences, Kreutzwaldi 5, 51006, Tartu, Estonia

**Keywords:** Lignocellulose, Agricultural biomass, Energy potential, Production waste, Zero-waste

## Abstract

This data article ranks 294 countries worldwide with more potential available, of cereal based agricultural residues for bioenergy production. Nine different cereal-based agricultural waste products (barley, wheat, millet, oat, rice, and rye straw, sorghum straw/stalk, and maize cob) are used. The tables and figures are grouped by the most prevalent Köppen-Geiger climate classification (tropical/megathermal, dry (desert and semi-arid), temperate/mesothermal, continental/microthermal), continent and region. The data was collected by the authors from FAO bioenergy and food security rapid appraisal tool (excel-based tools) that uses crop yields and production with 10 years (2005–2014) average annual production to estimate the residue yield (t/ha), by feedstock.

**Table:** Specifications table

Subject area	*Renewable energy, Agriculture*
More specific subject area	*Bioenergy, waste, residue yield, cereal*
Type of data	*Tables and figures*
How data was acquired	*The data were obtained from FAO bioenergy and food security rapid appraisal tool (Excel-based tools)*[1]
Data format	*Raw data were filtered and processed*
Experimental factors	*Yield (t/ha) of different cereal-based agricultural waste (barley, wheat, millet, oat, rice, and rye straw, sorghum straw/stalk, and maize cob) for bioenergy production*
Experimental features	*Aggregated by Köppen-Geiger climate classification,* continent and region
Data source location	*The data (from 294 countries worldwide) were collected from FAO bioenergy and food security rapid appraisal tool (Excel-based tools)*[1]
Data accessibility	*The raw data is available online at*http://www.fao.org/energy/bioenergy/bioenergy-and-food-security/assessment/befs-ra/natural-resources/en/
Related research article	*L. Rocha-Meneses, A. Ivanova, G. Atouguia, I. Ávila, M. Raud, K. Orupõld, and T. Kikas, “The effect of flue gas explosive decompression pretreatment on methane recovery from bioethanol production waste,” Industrial Crops and Products, vol. 127, pp. 66–72, 2019/01/01/2019.* *L. Rocha-Meneses, M. Raud, K. Orupõld, and T. Kikas, “Potential of bioethanol production waste for methane recovery,” Energy, vol. 173, pp. 133–139, 2019/04/15/2019.* *L. Rocha-Meneses, M. Raud, K. Orupõld, and T. Kikas, “Second-generation bioethanol production: A review of strategies for waste valorisation,” Agronomy Research, vol. 15, no. 3, pp. 830–847, 2017.*

**Table:** 

**Value of the Data** • Nowadays great attention has been paid to biorefinery concepts using lignocellulosic biomass as a feedstock. However, the costs of bioethanol production are still high mainly due to the energy input that is required to break down the plant cell well. Therefore, there is a search for alternative methods and feedstocks that will improve the energy output from the biomass [2]. • The production of energy in the form of methane has been reported as an effective method for the valorization of the lignocellulosic waste stream and as way of adding value to the bioethanol production chain [3], [4]. From the feedstock perspective, cereal based agricultural waste can also be utilized for bioethanol production. This solution can be used not only as a way of fully valorize all the waste streams from agricultural production, but also as a way of handle agricultural waste and minimize its impacts. • The dataset compiles 10-years average annual production of different cereal agricultural residues (at country level) to estimate its potential for bioenergy production. • The data can be used to study the different management strategies (at local level) for cereal agricultural waste, based on its potential, geographic location and climate classification. • Local scientific communities can study the best handling options (for the residues available in their region), that will increase the energy output from the biomass (e.g. biofuel or biogas production).

## 1 Data

**Fig. 1:**
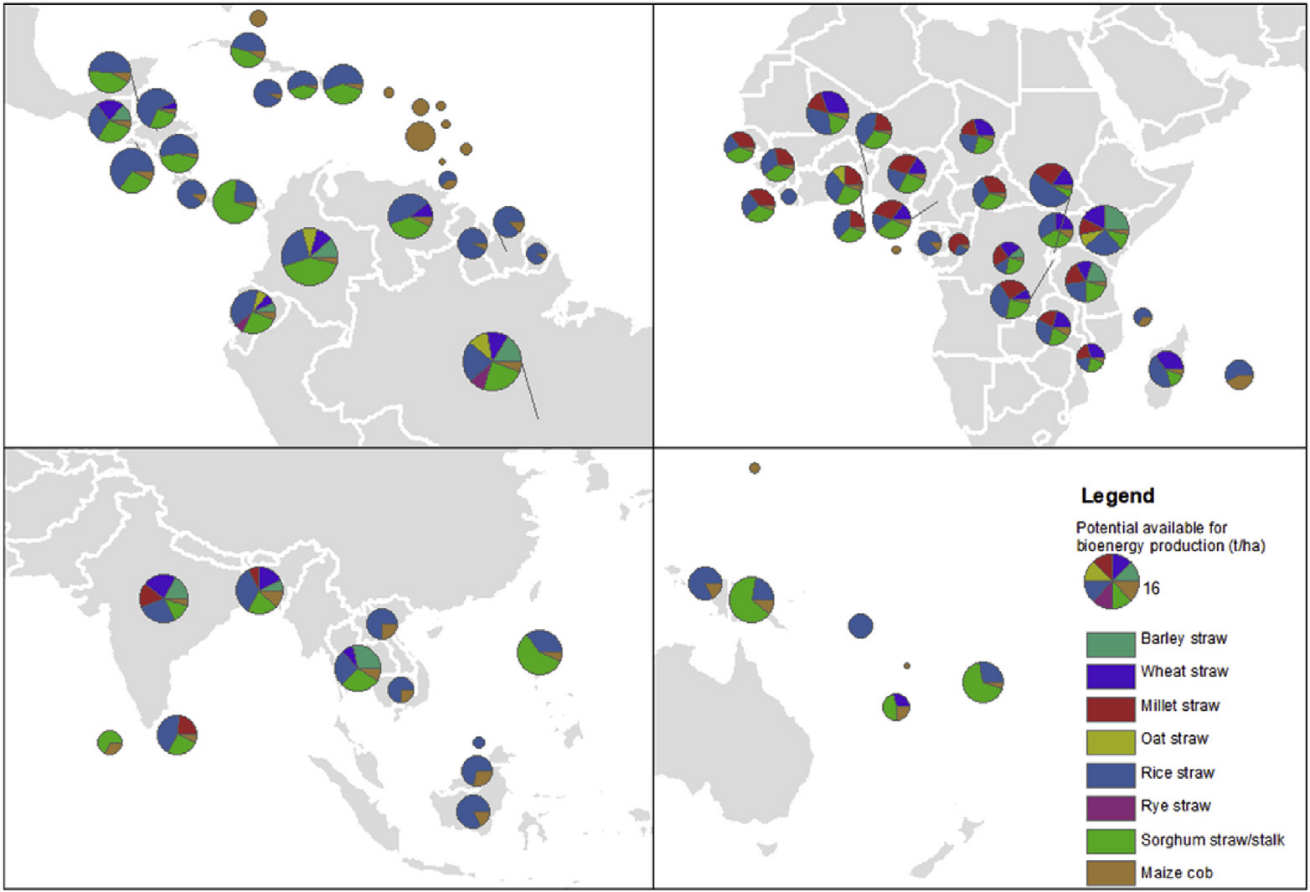
Potential available for bioenergy production (t/ha) (by Köppen-Geiger climate classification A, continent and region).

**Fig. 2:**
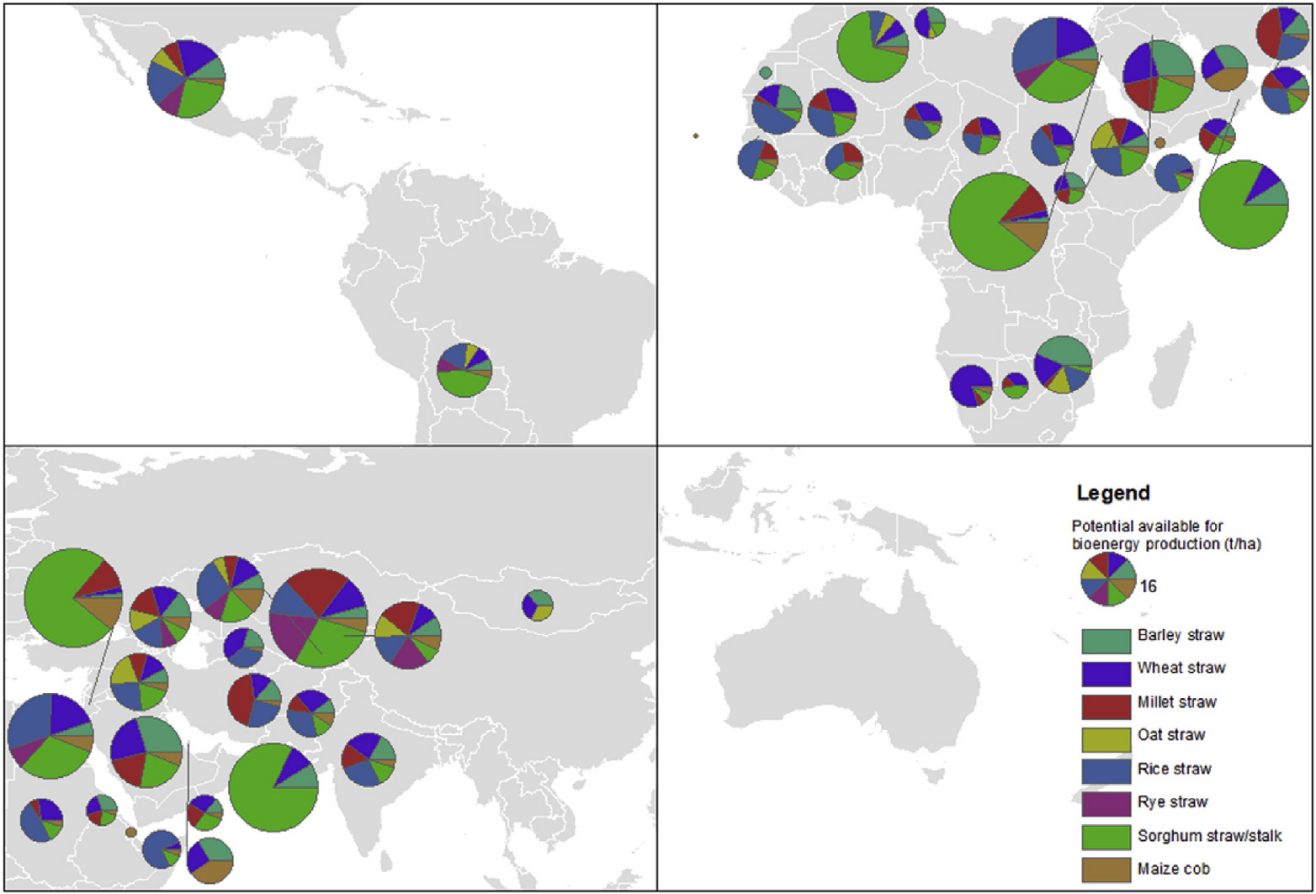
Potential available for bioenergy production (t/ha) (by Köppen-Geiger climate classification B, continent and region).

**Fig. 3:**
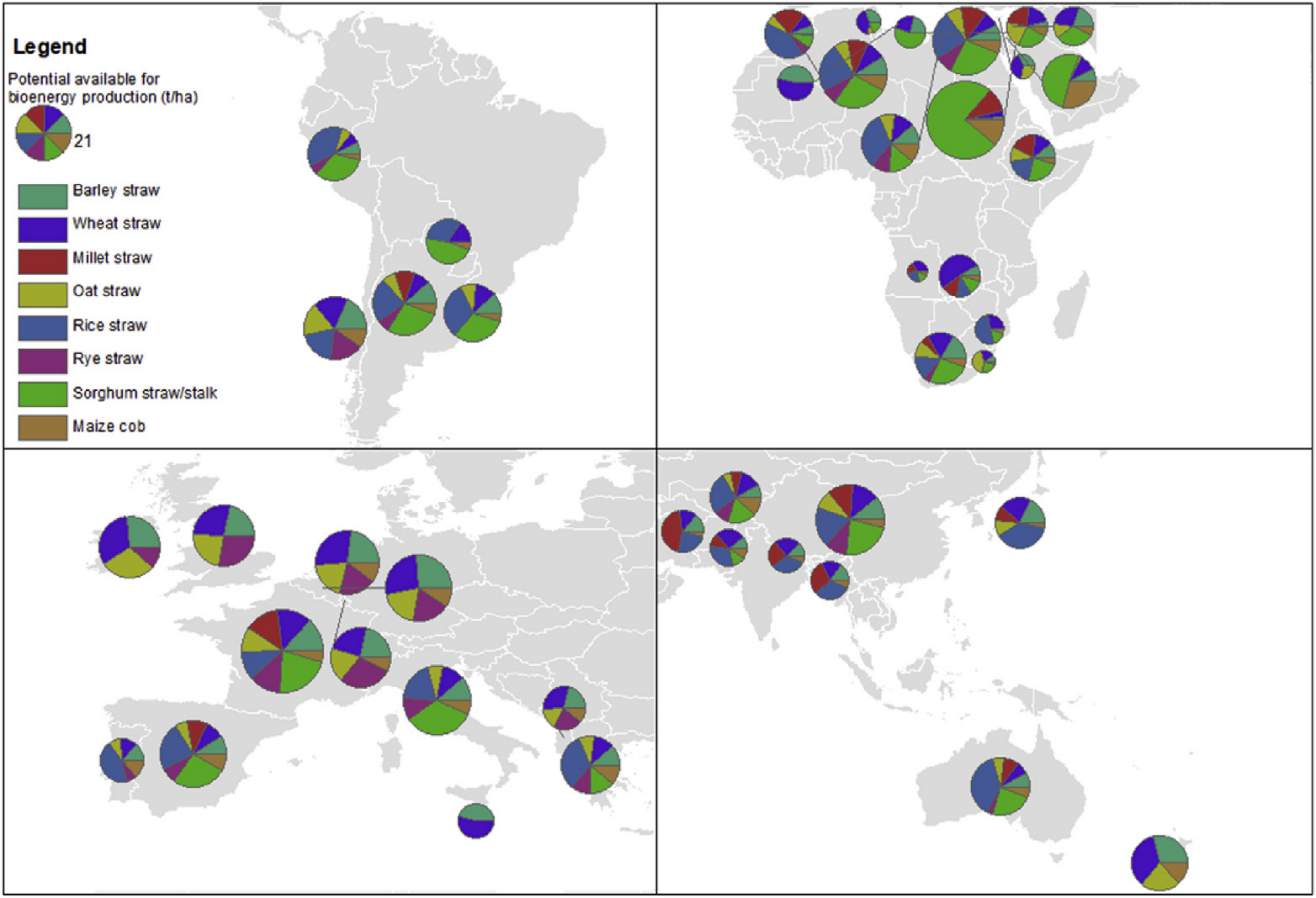
Potential available for bioenergy production (t/ha) (by Köppen-Geiger climate classification C, continent and region).

**Fig. 4:**
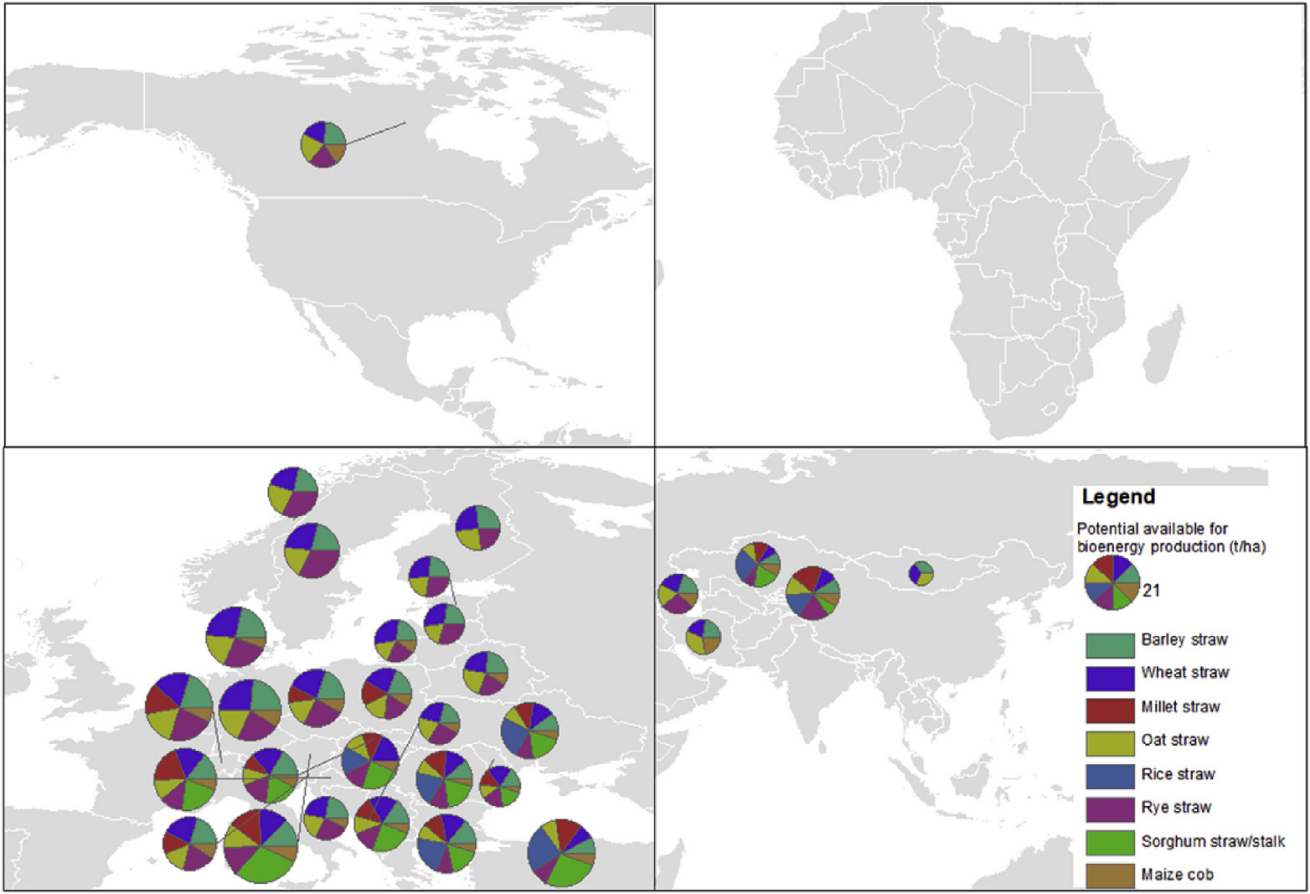
Potential available for bioenergy production (t/ha) (by Köppen-Geiger climate classification D, continent and region).

Due to the increase of the energy production mainly from non-renewable energy sources, there is a search for biobased solutions that will decrease the share of fossil fuels in the final energy mix. Biofuel or biogas production using cereal based agricultural waste as a feedstock can be used as a strategy to manage the large quantity of these residues. Table 1-Table 4 rank the countries (by continent) with the highest potential available for bioenergy, by Köppen-Geiger climate classification, continent and region. Fig. 1, Fig. 2, Fig. 3, Fig. 4 illustrate the potential available for bioenergy production (t/ha) by Köppen-Geiger climate classification and region. The total area of the pie charts is proportional to the overall amount of straw produced, meaning that bigger countries (with bigger production) have proportionally bigger pies.

**Table 1:** Ranking of countries in tropical/megathermal climates with more potential available (of cereal based agricultural waste) for bioenergy production (t/ha) (by Köppen-Geiger climate classification A, continent and region).

Africa	Barley straw	Wheat straw	Millet straw	Oat straw	Rice straw	Rye straw	Sorghum straw/stalk	Maize cob
**Equatorial**								
Benin	–	–	1.6	–	3.0	–	2.1	0.30
Burundi	–	0.72	2.0	–	3.0	–	2.0	0.27
Cameroon	–	1.2	2.5	–	1.3	–	2.7	0.50
Central African Republic	–	–	2.0	–	2.0	–	1.8	0.32
Chad	–	1.7	1.2	–	1.4	–	1.4	0.27
Comoros	–	–	–	–	1.2	–	–	0.62
Congo	–	–	1.6	–	0.58	–	–	0.22
Côte d'Ivoire	–	–	1.5	–	2.9	–	1.2	0.50
Democratic Republic of the Congo	0.68	1.2	1.3	–	0.73	–	1.3	0.19
Equatorial Guinea	–	–	–	–	–	–	–	–
Gabon	–	–	–	–	2.7	–	–	0.40
Gambia	–	–	1.7	–	1.1	–	1.8	0.25
Ghana	–	–	1.9	0.87	2.4	–	2.0	0.42
Guinea	–	–	1.7	–	1.9	–	2.1	0.33
Guinea-Bissau	–	–	2.2	–	1.7	–	1.8	0.28
Kenya	3.3	2.4	1.4	1.1	3.4	–	1.4	0.40
Liberia	–	–	–	–	1.2	–	–	–
Mali	–	3.0	1.6	–	3.1	–	1.7	0.54
Nigeria	–	1.3	2.3	–	1.7	–	2.2	0.44
Rwanda	–	1.5	2.4	–	5.0	–	0.57	0.41
Sao Tome and Principe	–	–	–	–	–	–	–	0.37
Seychelles	–	–	–	–	–	–	–	–
Sierra Leone	–	–	2.1	–	1.6	–	1.9	0.34
South Sudan	–	–	–	–	–	–	–	–
Togo	–	–	1.4	–	2.1	–	1.8	0.29
Uganda	–	1.6	–	–	2.0	–	2.1	0.54
United Republic of Tanzania	1.8	1.2	1.6	–	2.1	–	1.9	0.33
Southern								
Madagascar	–	2.3	–	–	2.8	–	0.94	0.37
Malawi	–	1.4	1.3	–	1.8	–	1.4	0.49
Mauritius	–	–	–	–	2.6	–	–	1.9
Mayotte	–	–	–	–	–	–	–	–
Mozambique	–	1.2	0.88	–	0.78	–	1.0	0.22
Réunion	–	–	–	–	3.9	–	–	2.1
Timor-Leste	–	–	–	–	2.4	–	–	0.46

Asia								

**Southern**								
Bangladesh	0.88	2.25	0.84	–	4.20	–	2.56	1.49
India	2.17	2.77	2.03	–	3.39	–	1.66	0.58
Maldives	–	–	–	–	–	–	2.06	0.98
Sri Lanka	–	–	2.06	–	3.75	–	2.19	0.63
**Far East**								
Brunei Darussalam	–	–	–	–	0.80	–	–	–
Cambodia	–	–	–	–	2.82	–	–	0.96
Indonesia	–	–	–	–	4.91	–	–	1.05
Lao People's Democratic Republic	–	–	–	–	3.74	–	–	1.24
Malaysia	–	–	–	–	3.64	–	–	1.48
Myanmar	–	1.65	1.77	–	3.89	–	1.71	0.86
Philippines	–	–	–	–	3.74	–	6.20	0.65
Singapore	–	–	–	–	–	–	–	–
Thailand	3.28	1.00	–	–	3.00	–	3.36	1.02
Vietnam	–	–	2.10	–	5.30	–	–	0.99

America								

**Northern**								
America Samoa	–	–	–	–	–	–	–	–
Anguilla	–	–	–	–	–	–	–	–
Saint Pierre and Miquelon	–	–	–	–	–	–	–	–
Turks and Caicos Islands	–	–	–	–	–	–	–	–
**Central**								
Antigua and Barbuda	–	–	–	–	–	–	–	0.47
Bahamas	–	–	–	–	–	–	–	1.46
Barbados	–	–	–	–	–	–	–	0.70
Belize	–	–		–	4.49	–	4.03	0.70
Cayman Islands	–	–	–	–	–	–	–	–
Costa Rica	–	–	–	–	3.78	–	–	0.51
Cuba	–	–	–	–	2.97	–	2.75	0.55
Dominica	–	–	–	–	–	–	–	0.37
Dominican Republic	–	–	–	–	4.53	–	3.27	0.37
El Salvador	–	–	–	–	6.77	–	2.92	0.74
Grenada	–	–	–	–	–	–	–	0.24
Guadeloupe	–	–	–	–	–	–	–	–
Guatemala	1.30	2.13	–	–	2.97	–	2.86	0.49
Haiti	–	–	–	–	2.43	–	1.73	0.19
Honduras	–	0.45	–	–	5.32	–	2.15	0.40
Jamaica	–	–	–	–	3.70	–	–	0.30
Martinique	–	–	–	–	–	–	–	–
Montserrat	–	–	–	–	–	–	–	1.52
Nicaragua	–	–	–	–	4.06	–	3.46	0.35
Panama	–	–	–	–	2.58	–	7.54	0.44
Puerto Rico	–	–	–	–	–	–	–	0.45
Saint Kitts and Nevis	–	–	–	–	–	–	–	–
Saint Lucia	–	–	–	–	–	–	–	–
Saint Vincent and the Grenadines	–	–	–	–	–	–	–	4.71
Trinidad and Tobago	–	–	–	–	1.05	–	–	0.61
Virgin Islands	–	–	–	–	–	–	–	–
**Southern**								
Brazil	2.90	2.22	–	2.02	4.36	1.56	4.44	1.04
Colombia	1.92	1.67	–	1.44	4.44	–	7.00	0.69
Ecuador	0.69	0.80	–	0.83	4.09	0.77	2.85	0.61
French Guiana	–	–	–	–	1.90	–	–	0.24
Guyana	–	–	–	–	4.38	–	–	0.34
Suriname	–	–	–	–	4.19	–	0.00	0.60
Venezuela	–	1.11	–	–	4.87	–	3.83	0.86

Oceania								

**Other islands**								
Cook Islands	–	–	–	–	–	–	–	–
Fiji	–	–	–	–	2.53	–	6.18	0.47
French Polynesia	–	–	–	–	–	–	–	–
Guam	–	–	–	–	–	–	–	0.62
Kiribati	0.00	–	–	–	–	–	–	–
Marshall Islands	–	–	–	–	–	–	–	–
Micronesia	–	–	–	–	1.43	–	2.89	0.35
Nauru	–	–	–	–	–	–	–	–
New Caledonia	–	1.12	–	–	–	–	1.76	0.92
Niue	–	–	–	–	–	–	–	–
Northern Mariana Islands	–	–	–	–	–	–	–	–
Palau	–	–	–	–	–	–	–	–
Papua New Guinea	–	–	–	–	2.45	–	7.47	1.23
Samoa	–	–	–	–	–	–	–	–
Solomon Islands	–	–	–	–	3.12	–	–	–
Tokelau	–	–	–	–	–	–	–	–
Tonga	–	–	–	–	–	–	–	–
Tuvalu	–	–	–	–	–	–	–	–
Vanuatu	–	–	–	–	–	–	–	0.14
Wallis and Futuna Islands	–	–	–	–	–	–	–	–

**Table 2:** Ranking of countries in dry (desert and semi-arid) regions with more potential available (of cereal based agricultural waste) for bioenergy production (t/ha) (by Köppen-Geiger climate classification B, continent and region).

Africa	Barley straw	Wheat straw	Millet straw	Oat straw	Rice straw	Rye straw	Sorghum straw/stalk	Maize cob
**Northern**								
Algeria	1.4	1.5	–	1.2	1.7	–	15	0.83
Cape Verde	–	–	–	–	–	–	–	0.05
Egypt	1.7	6.2	–	–	9.7	2.4	10	1.9
Eritrea	1.2	1.0	0.73	–	–	–	0.86	0.20
Libya	0.50	0.91	2.2	–	–	–	–	0.53
Mauritania	2.4	1.8	0.45	–	5.0	–	0.77	0.17
Sudan	–	2.2	0.56	–	3.7	–	1.1	0.37
Tunisia	1.2	1.8	–	0.30	–	–	0.76	–
Western Sahara	0.57	–	–	–	–	–	–	–
**Equatorial**								
Burkina Faso	–	–	1.6	–	2.1	–	1.9	0.41
Chad	–	1.7	1.2	–	1.4	–	1.4	0.27
Djibouti	–	–	–	–	–	–	–	0.43
Mali	–	3.0	1.6	–	3.1	–	1.7	0.54
Niger	–	1.9	0.89	–	2.1	–	0.67	0.21
Senegal	–	–	1.3	–	3.4	–	1.6	0.39
Somalia	–	0.37	–	–	4.6	–	0.82	0.33
**Southern**								
Botswana	–	1.1	0.46	–	–	–	1.3	0.05
Namibia	–	6.1	0.41	–	–	–	0.56	0.50
Saint Helena, Ascension and Tristan da Cunha	–	–	–	–	–	–	–	–
Zimbabwe	6.1	2.7	0.39	2.0	2.1	–	0.57	0.18

Asia								

**Central**								
Kyrgyzstan	1.87	2.01	3.86	2.21	3.14	3.65	1.46	1.50
Tajikistan	1.50	2.43	1.30	1.10	5.01	1.75	3.32	2.30
Turkmenistan	1.38	2.59	–	–	2.30	–	–	0.27
Uzbekistan	1.81	4.38	9.26	–	4.96	7.75	12.08	1.91
**Southern**								
Afghanistan	1.68	1.70	5.35	–	2.98	–	–	0.53
India	2.17	2.77	2.03	–	3.39	–	1.66	0.58
Pakistan	0.95	2.56	1.15	–	2.94	–	1.13	0.90
**Western**								
Azerbaijan	2.32	2.42	2.80	2.00	2.79	1.45	1.30	1.20
**Middle East**								
Bahrain	–	–	–	–	–	–	–	–
Iran	1.83	1.70	0.00	2.63	0.00	–	–	1.79
Iraq	1.09	1.71	1.60	2.77	3.57	–	2.63	0.71
Jordan	0.65	1.02	4.22	–	–	–	31.80	4.63
Kuwait	3.43	2.73	–	–	–	–	–	7.65
Oman	3.13	3.09	–	–	–	–	28.20	–
Qatar	3.04	2.32	–	–	–	–	–	3.73
Saudi Arabia	6.68	5.29	4.07	–	–	–	4.87	1.33
United Arab Emirates	7.89	4.57	–	–	–	–	102.62	6.32
Yemen	0.76	1.57	1.30	–	–	–	1.55	0.36
**Far East**								
Mongolia	1.44	1.24	–	1.30	–	–	–	–

America								

**Northern**								
Mexico	2.51	5.00	1.85	1.78	5.02	2.45	6.77	0.78
**Southern**								
Aruba	–	–	–	–	–	–	–	–
Bolivia	0.87	1.11	–	1.01	2.39	1.11	5.69	0.63

**Table 3:** Ranking of countries in temperate/mesothermal regions with more potential available (of cereal based agricultural waste) for bioenergy production (t/ha) (by Köppen-Geiger climate classification C, continent and region).

Africa	Barley straw	Wheat straw	Millet straw	Oat straw	Rice straw	Rye straw	Sorghum straw/stalk	Maize cob
**Northern**								
Morocco	1.0	1.5	3.7	1.1	7.1	0.93	1.5	0.19
Tunisia	1.2	1.8	–	0.30	–	–	0.76	–
**Equatorial**								
Ethiopia	1.6	1.8	2.8	1.5	2.7	–	3.5	0.65
**Southern**								
Angola	–	1.0	0.48	–	0.79	–	0.49	0.18
Lesotho	0.33	0.68	–	1.6	–	–	0.78	0.15
South Africa	3.1	3.0	1.0	1.9	2.7	0.79	4.9	1.0
Swaziland	–	1.6	–	–	3.0	–	0.85	0.29
Zambia	1.0	6.4	1.6	–	1.5	–	1.3	0.59

Asia								

**Central**								
Tajikistan	1.50	2.43	1.30	1.10	5.01	1.75	3.32	2.30
**Southern**								
Afghanistan	1.68	1.70	5.35	–	2.98	–	–	0.53
Bhutan	1.44	1.80	2.93	–	3.24	–	–	0.65
Nepal	1.11	2.12	2.12	–	2.92	–	–	0.55
Pakistan	0.95	2.56	1.15	–	2.94	–	1.13	0.90
**Middle East**								
Iran	1.83	1.70	0.00	2.63	0.00	–	–	1.79
Israel	1.59	1.91	–	0.42	–	–	11.01	5.89
Jordan	0.65	1.02	4.22	–	–	–	31.80	4.63
Lebanon	2.19	3.03	–	1.21	–	–	3.52	0.83
Palestine	1.45	1.65	–	–	–	–	3.88	–
Syrian Arab Republic	0.58	2.14	2.66	2.40	–	–	2.76	0.95
Turkey	2.50	2.37	4.09	2.42	7.69	2.86	8.61	1.86
**Far East**								
China	3.70	4.59	3.91	2.95	6.53	3.51	7.79	1.39
Japan	3.18	3.65	1.76	1.85	6.60	–	–	0.64

America								

**Northern**								
Bermuda	–	–	–	–	–	–	–	–
**Southern**								
Argentina	3.32	2.64	2.99	1.99	6.56	1.81	8.33	1.65
Chile	5.17	4.80	–	4.82	5.40	4.83	–	2.64
Paraguay	–	2.18	–	–	4.60	–	6.60	0.77
Peru	1.37	1.36	–	1.18	7.26	1.16	6.48	0.75
Uruguay	2.77	2.88	–	1.92	7.68	–	7.55	1.10

Europe								

**Northern**								
Faeroe Islands	–	–	–	–	–	–	–	–
**Southern/Southeast**								
Albania	2.78	3.68	–	2.03	–	2.83	–	1.44
Andorra	–	–	–	–	–	–	–	–
Cyprus	1.36	1.78	–	1.16	–	–	–	–
Gibraltar	–	–	–	–	–	–	–	–
Greece	2.69	2.63	–	2.08	7.52	2.66	3.34	2.64
Italy	3.71	3.61	–	2.53	6.40	3.50	11.06	2.27
Malta	4.08	4.70	–	–	–	–	–	–
Portugal	1.72	1.59	–	1.24	5.71	1.10	–	1.69
San Marino	–	–	–	–	–	–	–	–
Spain	2.80	2.87	3.11	2.02	7.35	2.34	8.35	2.59
**Western**								
Belgium	8.03	8.32	–	5.89	–	5.58	–	2.84
France	6.44	6.66	6.36	4.78	5.39	5.81	10.52	2.03
Ireland	7.08	8.44	–	7.81	–	3.05	–	–
Luxembourg	5.37	5.83	–	4.86	–	7.04	–	1.94
Monaco	–	–	–	–	–	–	–	–
Netherlands	6.51	8.20	–	5.73	–	5.34	–	2.97
United Kingdom	5.91	7.44	–	6.04	–	7.49	–	–

Oceania								

**Australasia**								
Australia	1.91	1.61	1.98	1.54	9.08	0.72	5.58	1.46
New Zealand	6.32	7.79	–	5.21	–	–	–	2.73
**Other islands**								
Norfolk Island	–	–	–	–	–	–	–	–

**Table 4:** Ranking of countries in continental/microthermal regions with more potential available (of cereal based agricultural waste) for bioenergy production (t/ha) (by Köppen-Geiger climate classification D, continent and region).

Asia	Barley straw	Wheat straw	Millet straw	Oat straw	Rice straw	Rye straw	Sorghum straw/stalk	Maize cob
**Northern**								
Russian Federation	2.05	2.06	2.14	1.71	4.88	2.13	2.18	0.96
**Central**								
Kazakhstan	1.25	1.04	1.54	1.29	3.61	1.25	2.59	1.19
Kyrgyzstan	1.87	2.01	3.86	2.21	3.14	3.65	1.46	1.50
**Western**								
Armenia	2.30	2.38	–	2.16	–	2.97	–	1.35
**Middle East**								
Iran (Islamic Republic of)	1.83	1.70	0.00	2.63	0.00	–	–	1.79
Turkey	2.50	2.37	4.09	2.42	7.69	2.86	8.61	1.86
**Far East**								
Democratic People's Republic of Korea	2.14	1.98	1.97	1.77	4.51	1.63	2.74	0.87
Mongolia	1.44	1.24	–	1.30	–	–	–	–
Republic of Korea	2.98	3.30	2.26	–	6.76	–	2.89	1.22

America								

**Northern**								
Canada	3.35	2.75	–	3.06	–	2.94	–	2.22
United States of America	3.69	2.82	2.98	2.44	7.96	2.07	7.30	2.36

Europe								

**Northern**								
Denmark	5.41	6.90	–	4.94	–	6.40	–	1.43
Estonia	2.79	3.06	–	2.46	–	3.29	–	–
Finland	3.66	3.65	–	3.52	–	3.19	–	–
Latvia	2.60	3.45	–	2.22	–	3.40	–	–
Lithuania	2.96	3.73	–	2.11	–	2.70	–	1.30
Norway	3.68	4.12	–	3.87	–	5.60	–	–
Sweden	4.44	5.74	–	4.13	–	6.84	–	–
**Central**								
Austria	4.92	4.95	5.56	4.21	–	4.88	11.77	2.55
Czech Republic	4.46	5.05	2.20	3.33	–	5.43	–	1.87
Germany	6.26	7.28	–	4.94	–	6.17	–	2.37
Hungary	–	4.04	2.64	2.64	3.53	2.91	5.18	1.54
Liechtenstein	–	–	–	–	–	–	–	–
Poland	3.36	3.94	2.86	2.72	–	3.11	–	1.53
Slovakia	3.66	4.05	2.04	2.24	–	3.62	4.25	1.56
Switzerland	6.38	5.66	4.87	5.41	–	7.25	–	2.29
**Southern/Southeast**								
Bosnia and Herzegovina	3.09	3.26	–	2.63	–	3.45	–	1.08
Bulgaria	3.38	3.44	2.59	1.88	5.36	2.17	3.97	1.23
Croatia	3.85	4.58	5.51	2.94	–	3.29	5.70	1.62
Montenegro	2.52	3.02	–	2.34	–	3.22	–	0.95
Romania	2.65	2.82	3.14	1.89	4.40	2.70	4.23	0.90
Serbia	3.39	3.68	2.66	2.36	–	2.84	5.36	1.28
Slovenia	4.17	4.47	2.70	2.97	–	3.94	–	1.90
Macedonia	2.96	2.98	3.02	1.89	5.87	2.74	2.45	1.07
**Eastern**								
Belarus	3.20	3.24	–	3.09	–	2.92	–	1.23
Republic of Moldova	1.84	2.29	1.67	1.28	–	2.01	2.01	0.69
Ukraine	2.33	2.95	2.55	1.98	5.31	2.45	4.02	1.24

## 2 Experimental design, materials, and methods

The data was obtained by the authors from FAO agricultural residues tool, which belongs to a series of Excel-based tools used to access the biomass potential of agricultural residues, wood fuel and wood residues, and crops.

The tables were grouped by the authors by Köppen-Geiger climate classification (A, B, C, and D), continent and region, using a simple excel spreadsheet.

The figures were drawn in the software ArcMap (version 10.6). For this, a worldwide base map was added to ArcMAP. The results were imported from the tables and pie charts were plotted (by region). The pie charts represent the potential of the different feedstocks available for bioenergy production.
